# Potential bioactive glycosylated flavonoids as SARS-CoV-2 main protease inhibitors: A molecular docking and simulation studies

**DOI:** 10.1371/journal.pone.0240653

**Published:** 2020-10-15

**Authors:** Sabri Ahmed Cherrak, Hafida Merzouk, Nassima Mokhtari-Soulimane

**Affiliations:** Laboratory of Physiology, Physiopathology and Biochemistry of Nutrition, Department of Biology, Faculty of Natural and Life Sciences, Earth and Universe, University of Tlemcen, Tlemcen, Algeria; University of Calgary, CANADA

## Abstract

A novel coronavirus responsible of acute respiratory infection closely related to SARS-CoV has recently emerged. So far there is no consensus for drug treatment to stop the spread of the virus. Discovery of a drug that would limit the virus expansion is one of the biggest challenges faced by the humanity in the last decades. In this perspective, to test existing drugs as inhibitors of SARS-CoV-2 main protease is a good approach. Among natural phenolic compounds found in plants, fruit, and vegetables; flavonoids are the most abundant. Flavonoids, especially in their glycosylated forms, display a number of physiological activities, which makes them interesting to investigate as antiviral molecules. The flavonoids chemical structures were downloaded from PubChem and protease structure 6LU7 was from the Protein Data Bank site. Molecular docking study was performed using AutoDock Vina. Among the tested molecules Quercetin-3-O-rhamnoside showed the highest binding affinity (-9,7 kcal/mol). Docking studies showed that glycosylated flavonoids are good inhibitors for the SARS-CoV-2 protease and could be further investigated by in vitro and in vivo experiments for further validation. MD simulations were further performed to evaluate the dynamic behavior and stability of the protein in complex with the three best hits of docking experiments. Our results indicate that the rutin is a potential drug to inhibit the function of Chymotrypsin-like protease (3CL pro) of Coronavirus.

## 1-Introduction

The novel coronavirus pneumonia is a contagious acute respiratory infectious disease caused by a novel virus strain, SARS-CoV-2 [1–6]. Since the outbreak of this disease in December 2019, the number of reported cases has surpassed thirty three million with more than a million deaths worldwide, as of September 28, 2020. Patients with the coronavirus pneumonia have symptoms such as dry cough, fatigue, dyspnea, difficulty breathing with fever, and high temperature. The severe acute respiratory syndrome coronavirus 2 (SARS-CoV-2) belongs to the coronaviruses family. Coronaviruses are single-stranded RNA enveloped viruses [7, 8].

The main encoded proteins of this RNA are a trimeric structural spike protein, a homodimeric protease [9, 10], an RNA polymerase and several nonstructural proteins [11, 12].

The virus is responsible of an elevated number of deaths as its transfer speed is higher than other coronaviruses. SARS-CoV-2 infection has then become a threat to public health. This disease caused by SARS-CoV-2 is affecting patients of a mild disease in most of them. However, in few cases, patients develop severe acute respiratory distress syndrome [13].

Until today, no specific vaccine has been developed to stop the spread of the virus and the need of a cure is now urgent. Viruses possess the ability to adapt and mutate quickly, leading research to find new therapeutic approaches. The main approach is to block the life cycle of the virus or to interfere in its fusion with the membrane so that the life cycle can be interrupted [14–17].

Another approach is the development of new drugs based on computational research tools. These tools are now fundamental to gain time and accuracy for developing new pharmacophores [18–20]. The process of identifying novel antiviral drugs to face the disease can rely on the screening of existing natural compounds known for their antiviral activity [21].

**Fig 1:**
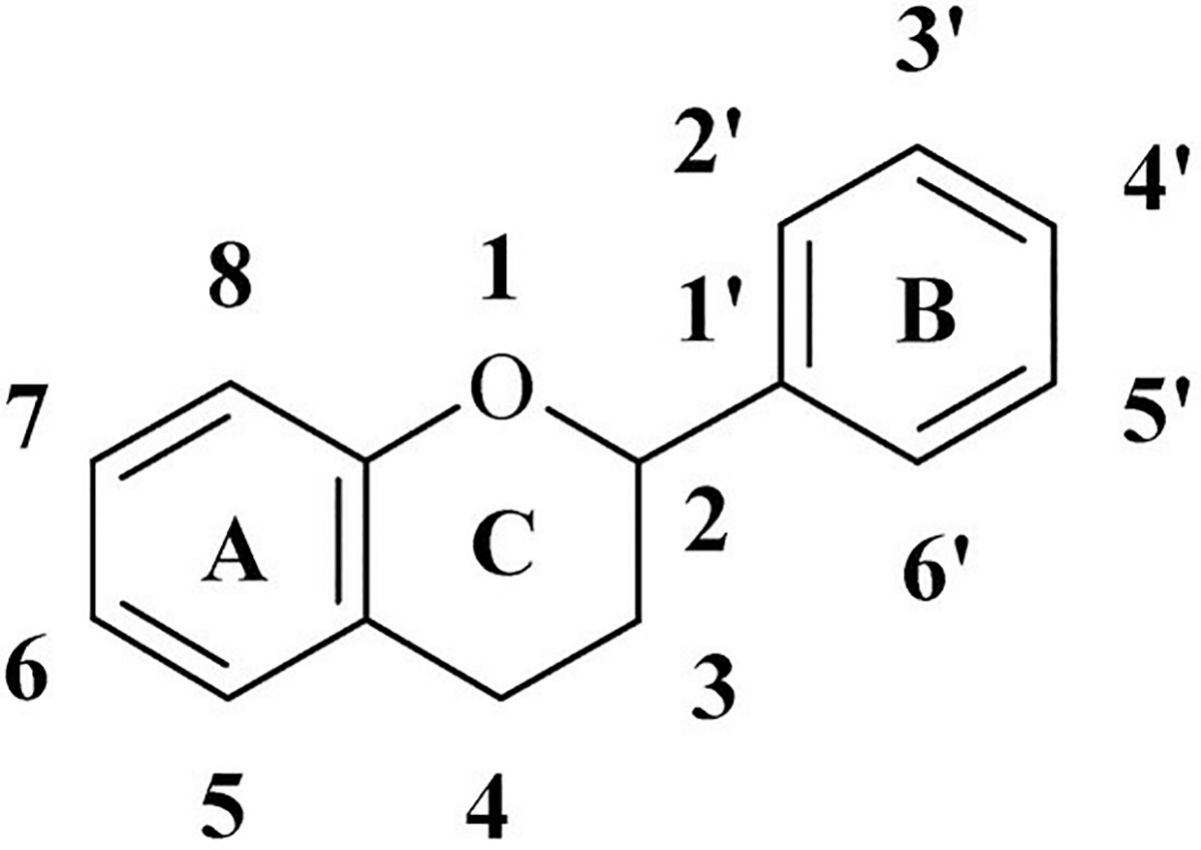
General flavonoid structure.

As flavonoids (Fig 1) are one of the most widely distributed plant compounds and play essential roles in plant physiology, they have been intensively investigated. In plants, flavonoids are mainly synthesized as aglycones, glycosides and methylated derivatives. They are biosynthesized through the phenylpropanoid pathway, transforming phenylalanine into 4-coumaroyl-CoA, which then enters the flavonoid biosynthesis pathway [22]. They possess a wide range of biological activities such as antioxidant [23–26], anticancer [27, 28], antibacterial [29, 30], antifungal [31] and antiviral activity [32–34]. Different classes of flavonoids display antiviral activities *in vitro* and *in vivo* specifically, apigenin, luteolin, quercetin [35, 36].

Flavonols, the most widely spread flavonoids (Fig 1) are characterized by a 3-hydroxy-2-phenylchromen-4-one backbone; and quercetin is the first representative compound and the most extensively investigated. Recent investigations also pointed out the antiviral activity of quercetin against a wide spectrum of influenza virus strains. It interacts with influenza hemagglutinin protein, thereby inhibiting viral-cell fusion [37]. Several other flavonols and derivatives have antivirals activities as a sulfated substituted rutin, showing significant activity against different HIV-1 isolates [38]. Several flavonoids are known by inhibiting virus proliferation either by stopping virus multiplication or by blocking cell entry. Indeed, several natural compounds including baicalin, scutellarin, hesperetin, nicotianamine and glycyrrhizin were predicted to have a capacity to binding ACE2 receptor with potential anti- SARS-CoV-2effects [36]. Moreover, quercetin, daidzein, puerarin, epigallocatechin, epigallocatechingallate, gallocatechingallate and kaempferol were reported to inhibit the proteolytic activity of SARS-CoV 3CL hydrolase [39]. Proteases and spike proteins are targets of choice for inhibition of SARS and MERS, and constant work is made to find novel molecules which can be able to interfere with virus life cycle either by computational methods or by experimental investigation [17, 40–42].

The SARS-CoV-2 main protease (Mpro), a chymotrypsine like hydrolase (3CL hydrolase) is an attractive target for anti-CoV drug design due to its responsibility for the maturation of main functional enzymes such as replicase and helicase [43–45].

In this work, a screening of natural flavonoids has been made and their potential inhibitory effect against SARS–CoV-2 Mpro by molecular docking and molecular dynamics has been tested. Indeed, our computed data suggest that strong interaction occur between flavonoids with sugar moieties and the main protease of the SARS-CoV-2virus. These results can represent a promising starting point for antiviral therapies that are alternative to the vaccination strategy.

## 2-Methods

### 2-1-Ligand and protein preparation

The three-dimensional structure of flavonoids were obtained from PubChem, in.sdf format and the structures were optimized using the Avogadro software and saved as mol2 files. The 3D structure of SARS-CoV2 main protease (Mpro, 3CLpro) (PDB ID: 6LU7) protein was obtained from Protein Data Bank (https://www.rcsb.org/). The resolution of the retrieved structure was 2.16 Å. The Mpro 3D structure was loaded into UCSF Chimera for molecular docking preparation [46].

### 2-2-Molecular docking

The inhibitory effect of thirty eight flavonoids on SARS-CoV-2 Mpro was studied by docking experiments using Autodock Vina software. The protease crystal structure consists of a 306 residues length chain with an excellent resolution of 2.16 Å co-crystallized with an engineered peptide inhibitor (N3) [47]. UCSF Chimera was used to remove all water molecules, heteroatoms, and co-crystallized solvent. The inhibitor was also abstracted from the PDB file and saved as a PDB file and used as positive control during docking studies. Finally, polar hydrogens and partial charges were added to the structure using UCSF Chimera point for antiviral therapies that are alternative to the vaccination strategy [46].

Molecular docking was finally performed by the AutoDock Vina [48] program in a rectangular box. The retained flavonoids were those with conformations of lower binding energy which are matching the active site defined by the co-crystallized N3 inhibitor.

### 2-3-Molecular dynamics simulation

Molecular dynamics simulations were performed with the GROMACS 2020 package using CHARMM36 force field installed on Linux system [49, 50]. The protein topology was prepared with the pdb2gmx tool. The protein complexes were solvated in a triclinic box.

Energy minimization of the system was performed by using the steepest descent algorithm [51]. Protein was solvated by TIP3 solvent model and 4 Na^+^ ions were introduced into the system to make the whole system neutral. The complexes consisting of the flavonoids and the Mpro were solvated in a water box with a minimal margin of 1.5 Å from any edge to any protein atom. An overall pressure and a temperature equal to 1 bar and 300 K were used with a time gap of 2 fs to stabilize the system. Temperature was kept constant inside the box, the v-rescale, an optimized Berendsen thermostat temperature coupling technique, was used. Parrinello-Rahman pressure coupling method was utilized in NPT equilibration. Energy minimization of the system was carried out by the steepest descent algorithm for 50,000 iteration steps and cut-off up to 1000 kJ.mol^-1^ to reduce the steric clashes. Particle mesh Ewald (PME) was employed to treat long-range electrostatics interactions with a Coulomb cut-off of 1.0 nm. The system was subjected to a final 50 ns molecular dynamic run each step of 2 fs.

### 2-4-Trajectory analysis and protein-ligand interaction energy analysis

Trajectory analysis was performed using various GROMACS analysis tools. The gmx rms, and gmx rmsf tools were respectively used to calculate the root mean square deviation (RMSD) and the root mean square fluctuations (RMSF) of protein. Hydrogen bonds were analyzed using gmx hbond tool, and the radius of gyration with the gyrate tool. Finally, plots were prepared using Grace Software.

## 3-Results and discussion

### 3-1-Molecular docking

The crystallographic structure of 3C like protease of SARS-CoV-2 (PDB id: 6LU7) in complex with a peptide-like inhibitor (N3) was made very recently available in the Protein Data Bank. From the above mentioned structure, we can clearly see a closed binding pocket around the peptide-like inhibitor.

**Fig 2:**
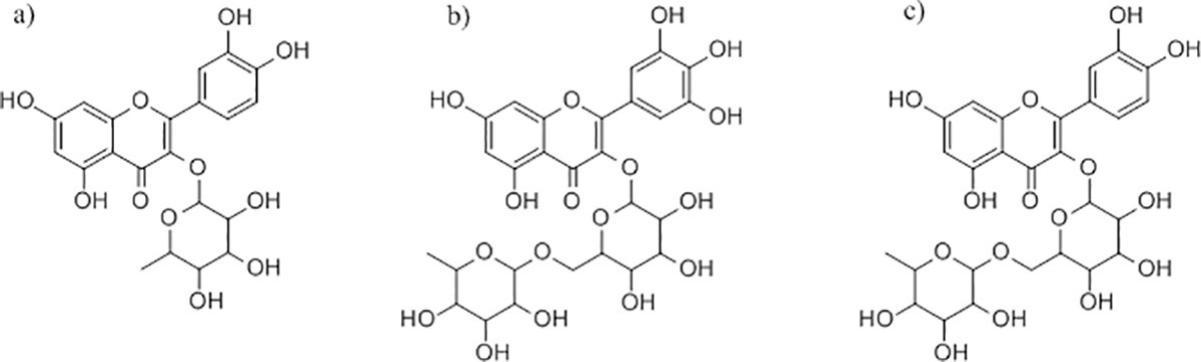
Structure of the compounds with highest score against Mpro active site a) Quercetin 3- Rhamnoside b) Myricetin 3-Rutinoside c) Rutin.

Docking of all flavonoid structures (Fig 2) was done against the active site of Mpro protein. Docking analysis provided several configurations that were scored to determine favorable binding modes. The flavonoid structures with the highest docking scores are summarized in Table 1. The three compounds with highest affinity (Fig 2) for the active site are quercetin 3-rhamonoside, myricetin 3-rutinoside and rutin with binding energies of -9.7, -9,3 and -9.2 kcal.mol^-1^ respectively. These values are higher than those obtained for the N3 inhibitor (-7.5 kcal.mol^-1^).

**Table 1:** Binding energies of flavonoids docked against main protease.

Structure Number	Name	Class	Substitution	Chemical formula	ΔG(Kcal.mol^-1^)
1	Quercetin 3-Rhamnose	Flavonol	3- Rhamnose	C_21_H_20_O_11_	-9,7
2	Myricetin 3-Rutinoside	Flavonol	3-Rutinoside	C_27_H_30_O_17_	-9,3
3	Rutin	Flavonol	3-Rutinoside	C_27_H_30_O_16_	-9,2
4	Myricitrin	Flavonol	3- Rhamnose	C_21_H_20_O_12_	-8,9
5	Quercetin3-O-Neohesperidoside	Flavonol	3-Rhamnose glucose	C_27_H_30_O_16_	-8,9
6	Myricetin 3-Galactoside	Flavonol	3- Galactose	C_21_H_20_O_13_	-8,7
7	Nicotiflorin	Flavonol	3-Rutinoside	C_27_H_30_O_15_	-8,7
8	Quercetin 3 4 ' di Glu	Flavonol	3,4' Glucose	C_27_H_30_O_17_	-8,7
9	Quercetin3-O-alpha-D-Arabinofuranoside	Flavonol	3-Arabinose	C_20_H_18_O_11_	-8,5
10	Hyperoside	Flavonol	3- Galactose	C_21_H_2_0O_12_	-8,4
11	Lonicerin	Flavone	7-Rutinoside	C_27_H_30_O_15_	-8,3
12	Naringenin 7-O-Rutinoside	Flavanone	7-Rutinoside	C_27_H_32_O_14_	-8,3
13	Apigenin7-O-neohesperidoside	Flavone	7-neohesperidoside		-8,2
14	Datiscin	Flavonol	3-Glucose	C_27_H_30_O_15_	-8,2
15	Hesperidin	Flavanone	7-Rutinoside	C_28_H_34_O_15_	-8,1
16	Isoquercitrin	Flavonol	3-Glucose	C_21_H_20_O_12_	-8,1
17	Naringin	Flavanone	7-Rutinoside	C_27_H_32_O_14_	-8,1
18	Narirutin	Flavanone	7-Rutinoside	C_27_H_32_O_14_	-8,1
19	Quercetin3Gal-7-Rhamnose	Flavonol	3- Galactose 7-Glucose	C_27_H_30_O_16_	-8,1
20	Quercetin3-O-Glucuronide	Flavonol	3-Glucuronide	C_21_H_18_O_13_	-8,1
21	QuercetinMalonyl Glucose	Flavonol	Glucose	C_24_H_22_O_15_	-8
22	Spireaoside	Flavonol	4' Glucose	C_21_H_20_O_12_	-8
23	EGCG	Flavanol	-	C_22_H_18_O_11_	-7,9
24	Sylibin B		-	C_25_H_22_O_10_	-7,9
25	Kaempferol3-O-Rhamnoside	Flavonol	3-Rhamnose	C_21_H_19_O_10_	-7,7
26	Epicatechine 3'-glucuronide	Flavanol	3'-Glucuronide	C_21_H_22_O_12_	-7,6
27	Fisetin	Flavonol	-	C_15_H_10_O_6_	-7,6
28	Prunin	Flavanone	7-Glucose	C_21_H_22_O_10_	-7,6
29	**N3 Inhibitor**	Peptide	-	C_35_ H_48_ N_6_ O_8_	-7,5
30	Genistin	Isoflavone	7-Glucose	C_21_H_20_O_10_	-7,5
31	Ladanein	Flavone	-	C_17_H_14_O_6_	-7,5
32	Myricetin 3- Glucose	Flavonol	3-Glucose	C_21_H_20_O_13_	-7,5
33	Quercetin	Flavonol	-	C_15_H_10_O_7_	-7,5
34	Icaritin	Flavonol	-	C_21_H_20_O_6_	-7,4
35	Myricetin	Flavonol	-	C_15_H_10_O_8_	-7,4
36	Isorhamnetin	Flavonol	-	C_16_H_12_O_7_	-7,3
37	Cyanidin	Anthocyanidol	-	C_15_H_11_O_6+_	-7,2
38	Diadzein	Isoflavone	-	C_15_H_10_O_4_	-7,2
39	Nobiletin	Flavone	polymethoxyl	C_21_H_22_O_8_	-6,5

Thirty eight flavonoids have been tested in this study by molecular docking against the active site of the SARS-CoV-2Mpro. The results which are summarized in Table 1 show that natural aglycone flavonoids possess high docking scores; with a maximum score achieved by EGCG with -7,9 kcal.mol^-1^.

**Fig 3:**
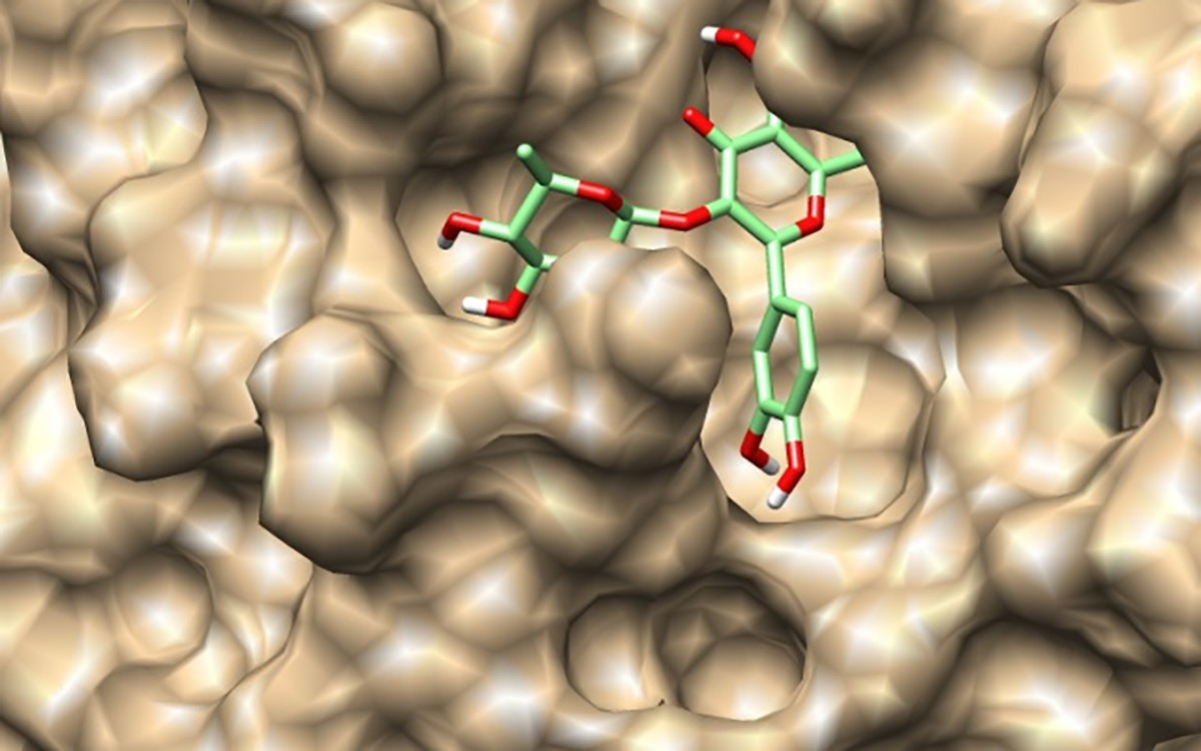
Binding pose of Quercetin -3 rhamnoside in the active site of SARS-CoV-2main protease.

This value is comparable to the score obtained for N3 inhibitor co-crystallized with the protease (6LU7), used here as a positive control (-7,7 kcal/mol). Furthermore, we can clearly see that glycosylated flavonoids display the highest scores; with a maximum value of 9,7 kcal.mol^-1^ for quercetin 3-rhamnoside. As shown in Fig 3, quercetin 3-rhamnoside is well installed in the active site and the position is matching with the N3 peptide inhibitor.

The best compounds possess sugar moieties in their structure, and the position seems to be important. Compounds substituted at the carbon 3 display higher scores than those substituted at the carbone 7. Genistin, a flavonoid substituted at position 7 displays low score (-7,5 kcal.mol^-1^) compared to datiscin or hyperoside (-8,2; -8,4 kcal.mol^-1^) among others.

The nature of the sugar moiety is also important to the activity.As we can see, the top 5 compounds are flavonols and they possess either a rhamnose or a rutinoside (a dissaccharide: Glucose-Rhamnose) at position 3. Quercetin 3-Rhamnoside (Fig 3) and myricetin 3-Rutinoside bind with more strength than datiscin which is substituted at position 3 with glucose.

However, low bioavailability of flavonoids is a major inconvenient to their use as natural drugs. The presence of sugar moieties usually leads to enhanced bioavailability of the respective flavonoid aglycone depending on the nature of the substituted sugar. Flavonoids with glucose moieties seems to be absorbed more rapidly than flavonoids with rhamnosides, rhamnoglucosides, rutinosides and neohesperidosides substitutions [52].

**Fig 4:**
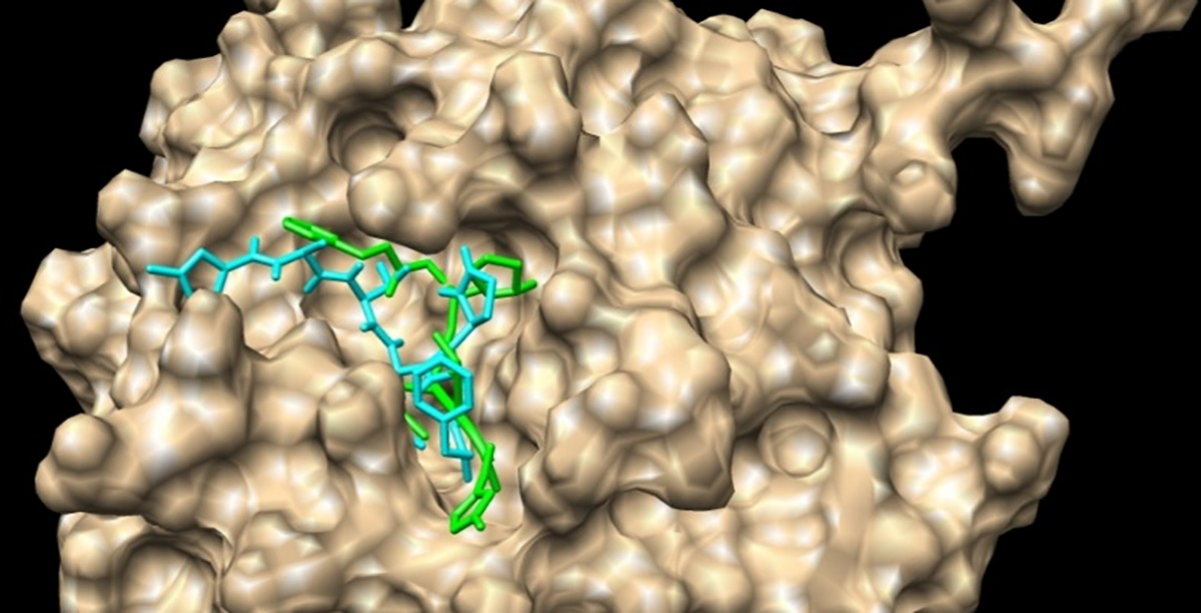
Binding pose of N3 inhibitor: Docked (green) co-crystallized (cyan) at the active site of the SARS-CoV-2Mpro (superimposed structures).

Fig 4 shows the binding poses of the N3 peptide co-crystallized with SARS-CoV-2 Mpro and a superimposed structure of the same peptide is obtained by a docking tool.

It is assumed that bioavailability of glyco-flavonoids is achieved by aglycone part in blood plasma after the cleavage of the glycosidic part by hydrolyzing enzymes in the human gastrointestinal tract [53]. Glucose moieties are easily hydrolyzed in the small intestine epithelial cells. However, the lack of α- L -rhamnosidase or rutinosidase in human cells makes the bioavailability of relative flavonoids largely dependent on their hydrolysis by intestinal bacteria [53]. Furthermore, few intestinal bacterial strains can achieve cleavage of these types of bonds [54].

**Fig 5:**
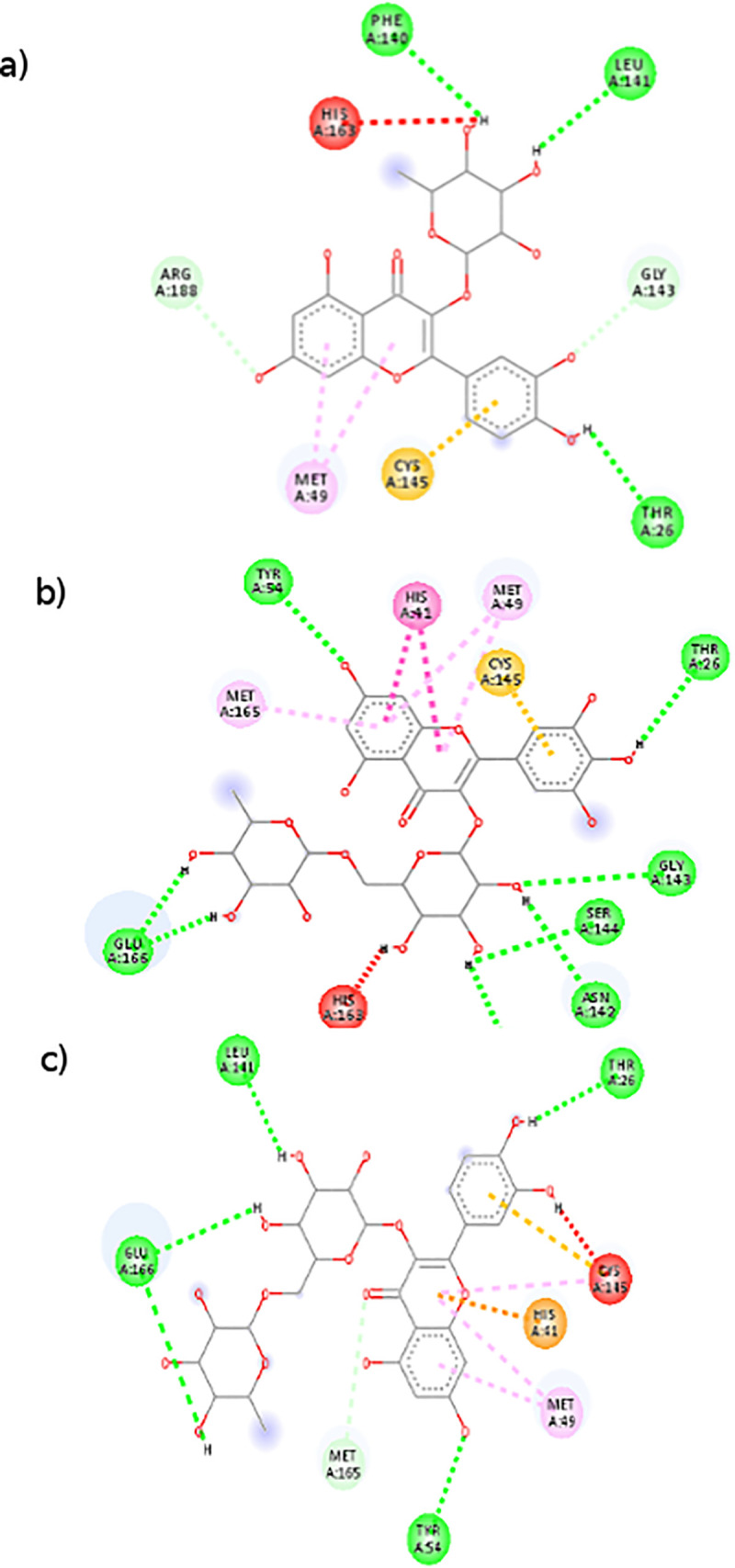
Interaction of flavonoids at the active site of the SARS-CoV-2Mpro a) Quercetin -3 Rhamnose, b) Myricetin 3-rutinoside, c) Rutin.

Discovery studio visualizer was further used to determine hydrogen bonds occurring between amino acids of the active site and flavonoids (Fig 5). The N3 inhibitor was docked to the SARS-CoV-2Mpro to be further compared to docked generated model with Autodock vina. The superimposed structures of the co-crystallized N3 inhibitor (Fig 4) are closely related indicating a good accuracy of the automated tool.

Results (Table 2) show that compounds that undertake bonds with the highest strength usually bind to Thr26, Ty54, His41, Asn142, Gly143, Cys145and Glu166 in the substrate binding subsite S1 Fig for the proteolytic activity [55, 56], leading to strong binding due to hydrogen bonds. Mpro consists of three domains and substrate binding site resides in a cleft between domain I and II, while domain III is involved in catalytic function. Dimerization of the protein is required for its catalytic regulation [57]. Glu166 residue is a key amino acid involved in the dimerization of Mpro and creation of substrate binding pocket [45, 58]. Cys141 and His41 residue forms a catalytic dyad on the active site of the protein essential for its catalytic function. Similar results were recently obtained by molecular docking with different molecules against the Mpro protein [57, 59, 60]. Quercetin 3-Rhamnose, a flavonol with a rhamnose at position 3 of the C ring, is involved in several hydrogen bonds contracted with Thr26, Phe140, Leu141 and Gly143 (Fig 5A).

**Table 2:** Interaction of the best hits of the docking experiments.

Best Docked compounds	AA	Interaction	Distance (A°)
**Quercetin 3-Rhamnose**	Thr26	Conventional H bonds	2.59
	Met49	Pi-Alkyl	4.47
	Phe140	Conventional H bonds	2.77
	Leu141	Conventional H bonds	2.60
	Gly143	C-H bond	2.54
	Cys145	Pi-Sulfur	4.94
	Arg188	C-H bond	2.72
**Myricetin 3-rutinoside**	Thr26	Conventional H bonds	2.49
	His41	Pi-Pi	4.58
	Met49	Pi-Alkyl	4.85
	Tyr54	Conventional H bonds	2.95
	Asn142	Conventional H bonds	2.77
	Gly143	Conventional H bonds	3.01
	Ser144	Conventional H bonds	2.93
	Met165	Pi-Alkyl	5.45
	Glu166	Conventional H bonds	2.40
**Rutin**	Thr26	Conventional H bonds	2.73
	His41	Pi-Cation	4.06
	Met49	Pi-Alkyl	4.41
	Tyr54	Conventional H bonds	2.73
	Leu141	Conventional H bonds	2.50
	Cys145	Pi-Sulfur	5.03
	Met165	C-H bond	2.80
	Glu166	Conventional H bonds	2.63

The second and third flavonoids with highest affinity, myrictin 3-Rutinoside and rutin are also flavonols but with a rutinoside (Glucose-Rhamnose) at the 3 position. Fig 5B and 5C show that both compounds display hydrogen bonds with Mpro leading to high affinity binding to the active site. Results show that glycosylated flavonols with a sugar moiety at position 3 with at least a rhamnose in their structure are the compounds that bind with higher affinity to the active site pocket of the Mpro.

### 3-2-Molecular dynamics simulations

Molecular dynamics simulations are of proven in-silico methods for obtaining dynamic data at atomic spatial resolution [2, 51]. The docked Mpro complexes with the three compounds with highest scores: Quercetin -3 Rhamnose, Myricetin 3-rutinoside and Rutin were subjected to MD simulations for 50 ns to analyze the stability of the complexes.

**Fig 6:**
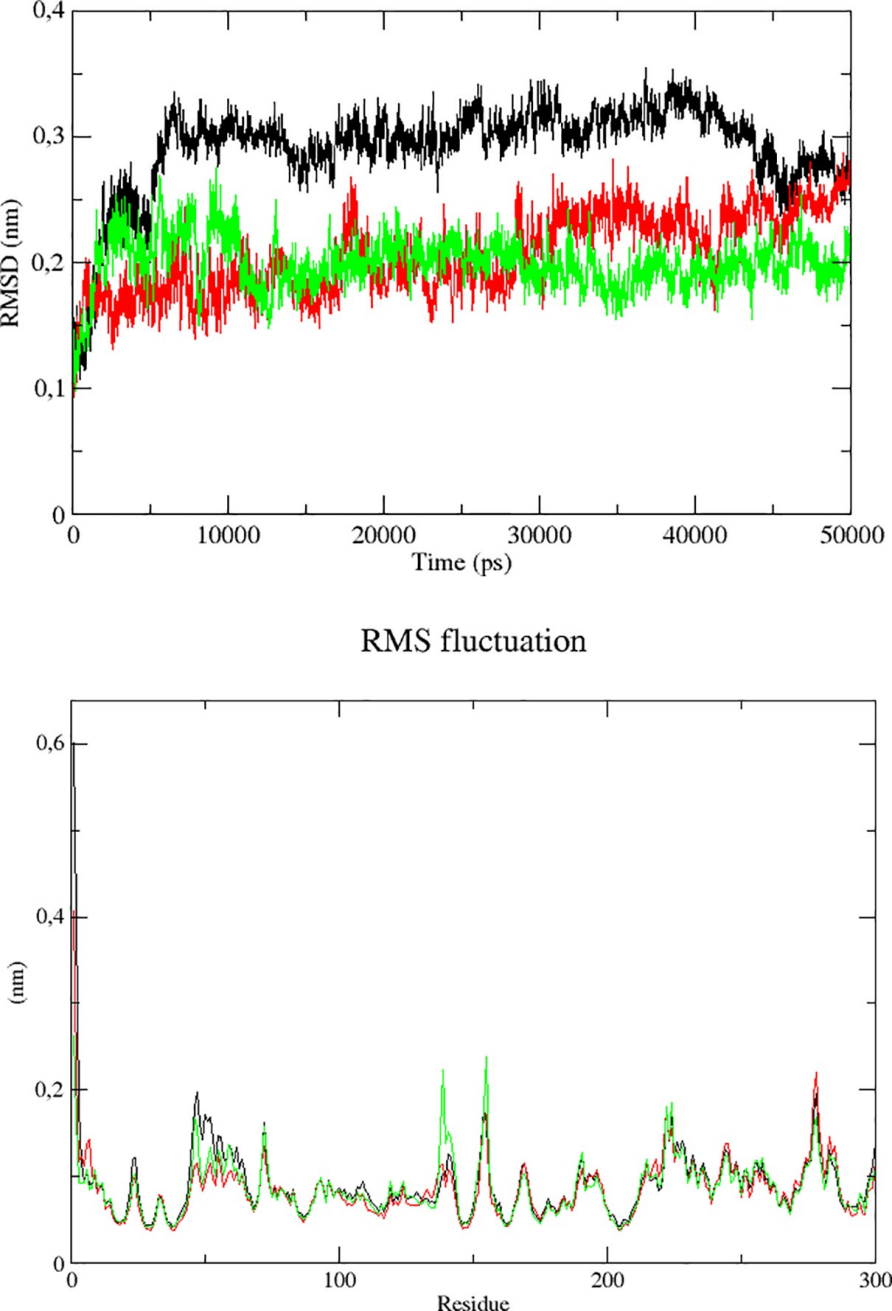
RMSD and RMSF plots for Mpro complex with quercetin -3 rhamnoside (black), myricetin 3-rutinoside (red) and rutin (green) for 50 ns.

RMSD plot is usually used to understand the stability of the complex while RMSF is used to understand the structural flexibility (Fig 6)

The Mpro in complex with rutin (green) showed the most stable RMSD during the 50 ns simulation around 0.2 nm. The M pro with compound Myricetin 3-rutinoside (red) is also stable and constant range of RMSD between 0.15 nm and 0.25 nm. Slight increase was observed after 40 ns simulation. The quercetin 3-rhamnose (black) showed stable RMSD between 5 and 40 ns with values close to 0.3 nm. After 40 ns RMSD value decreased to 0.25 nm (Fig 6). The differences of backbone RMSD in quercetin 3-Rhamnoside and myricetin 3-rutinoside indicate that M pro undergoes conformational changes.

RMSF trajectories provide important information regarding the stability of the complex. High fluctuations in the plot indicate more flexibility and unstable bonds. On the other side, low value or less fluctuation indicates well structured regions in the complex and less distortion. As depicted in Fig 6, all the systems showed almost similar patterns. The overall value of RMSF for all the three complexes was around 0.1 nm as clearly seen in Fig 6.

**Fig 7:**
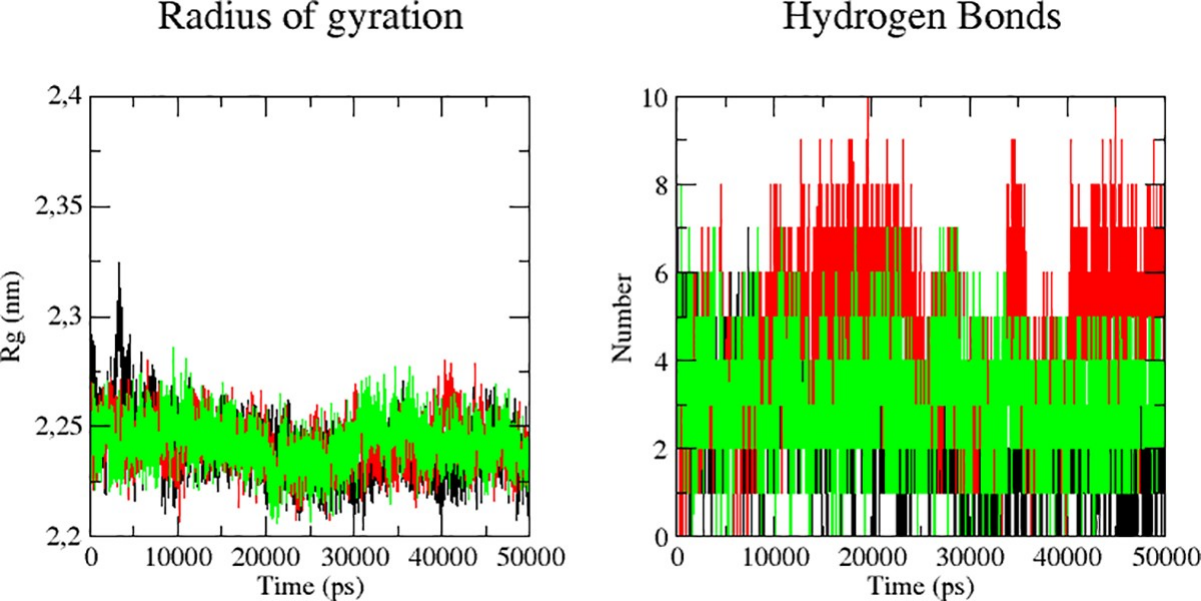
Rg and hydrogen bonds plots for Mpro complex with quercetin -3 rhamnose (black), myricetin 3-rutinoside (red) and rutine (green) for 50 ns.

In order to determine the compactness of the system, Rg was plotted against time. Higher Rg values illustrate less compactness with conformational entropy, while low Rg values are explained as high stability in the structure (more folded). As shown in Fig 7, the simulation Rg values of the three compounds are in range 0.22–0.23 nm.

The simulation Rg values of the three compounds reported as 2.2–2.3 nm. The fewer changes in the Rg value showed the stability of the protein in the complex suggesting that the binding of these three molecules does not induce structural changes. The Rg range of the three complexes (Fig 7) support their condensed architecture as well as size. Moreover; potential and kinetic energy plots confirm the stability of the complexes (S1 Fig). Hydrogen bonding plays an essential role in determining the binding strength between ligands and protein. Rutin (green) id (black) has constant range of hydrogen bond between 2 to 4 in whole simulation while quercetin 3-rhamnose and myricetin 3-rutinoside showed changes in bonding. More hydrogen(>6) bonds between 10 to 25 ns for myricetin 3-rutinoside, suggesting a conformational change in the binding site during simulation (Fig 7). Over all, observation suggested that Mpro protein in complex with the three compounds is stable during simulation. However, it is clear that rutin complex is more stable and therefore a better candidate as potential drug for inhibiting the Mpro protein.

## Conclusion

Until now, the spread of SARS-CoV-2has stimulated research to find an existing drug for a rapid treatment. At that time, the confirmed cases are over 7 million and rising, and the deaths are approaching 400000, and governments fear a second infection wave. Isolation strategy was mandatory to limit the spread of the virus, but solutions have to be found urgently.

The main protease taken into account as molecular target for stopping the spread of the virus is an interesting strategy.

Results show that glycosylated flavonoids display a strong inhibitory activity on SARS-CoV-2 Mpro. Glycosylated flavonoids seems to bind with more strength than the N3 co-crystallized inhibitor. The binding of the flavonoids at the protease active site is clearly structure dependant. Compounds binding with more strength are flavonols substituted with mono or disaccharides at position C3. The nature of sugar is also primordial to the activities, as flavonoids with a rhamnose possess the strongest binding affinity. As flavonoids with rhamnose moiety undergo no or little modification in the intestine, they might be a good candidate in the treatment of the SARS-CoV-2coronavirus.

## Supporting information

S1 Fig Kinetic and potential energies for Mpro complex with (a), Quercetin -3 Rhamnose (b) Myricetin 3-rutinoside and (c) Rutin for 50 ns.
(DOCX)

## List of abbreviations

AA: Amino Acids
3CL hydrolase: 3-chymotrypsin-like hydrolase
EGCG: Epigallocatechingallate
MERS: Middle East respiratory syndrome
Mpro: Main protease
SARS-CoV-2: Severe acute respiratory syndrome coronavirus-2

## References

[1] Kumar S, Rathi B. Coronavirus Disease COVID-19: A New Threat to Public Health. Current Topics in Medicinal Chemistry. 2020;20(8):599–600. 10.2174/1568026620999200305144319 32133964

[2] Kumar A, Choudhir G, Shukla SK, Sharma M, Tyagi P, Bhushan A, et al Identification of phytochemical inhibitors against main protease of COVID-19 using molecular modeling approaches. Journal of Biomolecular Structure and Dynamics. 2020;(just-accepted):1–21. 10.1080/07391102.2020.1772112 PMC7284142 32448034

[3] Hemida MG, Abduallah MMB. The SARS-CoV-2 outbreak from a one health perspective. One Health. 2020:100127 10.1016/j.onehlt.2020.100127 32292814 PMC7102578

[4] Sk MF, Roy R, Jonniya NA, Poddar S, Kar P. Elucidating biophysical basis of binding of inhibitors to SARS-CoV-2 main protease by using molecular dynamics simulations and free energy calculations. Journal of Biomolecular Structure and Dynamics. 2020;(just-accepted):1–21. 10.1080/07391102.2020.1768149 PMC7284146 32396767

[5] Ahmad S, Abbasi HW, Shahid S, Gul S, Abbasi SW. Molecular Docking, Simulation and MM-PBSA Studies of Nigella Sativa Compounds: A Computational Quest to identify Potential Natural Antiviral for COVID-19 Treatment. Journal of Biomolecular Structure and Dynamics. 2020;(just-accepted):1–16. 10.1080/07391102.2020.1775129 PMC7298883 32462996

[6] Kumar D, Kumari K, Jayaraj A, Kumar V, Kumar RV, Dass SK, et al Understanding the binding affinity of noscapines with protease of SARS-CoV-2 for COVID-19 using MD simulations at different temperatures. Journal of Biomolecular Structure and Dynamics. 2020:1–14. 10.1080/07391102.2020.1752310 PMC7212547 32362235

[7] CSG of the International CSG. The species Severe acute respiratory syndrome-related coronavirus: classifying 2019-nCoV and naming it SARS-CoV-2. Nature Microbiology. 2020:1. 10.1038/s41564-020-0695-z PMC7095448 32123347

[8] Umesh, Kundu D, Selvaraj C, Singh SK, Dubey VK. Identification of new anti-nCoV drug chemical compounds from Indian spices exploiting SARS-CoV-2 main protease as target. Journal of Biomolecular Structure and Dynamics. 2020;(just-accepted):1–9. 10.1080/07391102.2020.1763202 PMC7232883 32362243

[9] Phan T. Genetic diversity and evolution of SARS-CoV-2. Infection, Genetics and Evolution. 2020;81:104260 10.1016/j.meegid.2020.104260 32092483 PMC7106203

[10] Zhang L, Lin D, Sun X, Curth U, Drosten C, Sauerhering L, et al Crystal structure of SARS-CoV-2 main protease provides a basis for design of improved α-ketoamide inhibitors. Science. 2020;368(6489):409–12. 10.1126/science.abb3405 32198291 PMC7164518

[11] Nyakas M, Aamdal E, Jacobsen KD, Guren TK, Aamdal S, Hagene KT, et al Prognostic biomarkers for immunotherapy with ipilimumab in metastatic melanoma. Clinical & Experimental Immunology. 2019;197(1):74–82. 30821848 10.1111/cei.13283 PMC6591141

[12] Tai W, He L, Zhang X, Pu J, Voronin D, Jiang S, et al Characterization of the receptor-binding domain (RBD) of 2019 novel coronavirus: implication for development of RBD protein as a viral attachment inhibitor and vaccine. Cellular & molecular immunology. 2020:1–8. 10.1038/s41423-020-0400-4 PMC7091888 32203189

[13] Feng Z, Yu Q, Yao S, Luo L, Duan J, Yan Z, et al Early prediction of disease progression in 2019 novel coronavirus pneumonia patients outside Wuhan with CT and clinical characteristics. MedRxiv. 2020.

[14] Höfer C, Di Lella S, Dahmani I, Jungnick N, Bordag N, Bobone S, et al Structural determinants of the interaction between influenza A virus matrix protein M1 and lipid membranes. Biochimica et Biophysica Acta (BBA)-Biomembranes. 2019;1861(6):1123–34. 30902626 10.1016/j.bbamem.2019.03.013

[15] Bobone S, Hilsch M, Storm J, Dunsing V, Herrmann A, Chiantia S. Phosphatidylserine lateral organization influences the interaction of Influenza virus Matrix Protein 1 with lipid membranes. Journal of virology. 2017;91(12):e00267–17. 10.1128/JVI.00267-17 28356535 PMC5446629

[16] Khan MT, Ali A, Wang Q, Irfan M, Khan A, Zeb MT, et al Marine natural compounds as potents inhibitors against the main protease of SARS-CoV-2. A molecular dynamic study. Journal of Biomolecular Structure and Dynamics. 2020;(just-accepted):1–14. 10.1080/07391102.2020.1769733 PMC7284144 32410504

[17] De Oliveira OV, Rocha GB, Paluch AS, Costa LT. Repurposing approved drugs as inhibitors of SARS-CoV-2 S-protein from molecular modeling and virtual screening. Journal of Biomolecular Structure and Dynamics. 2020;(just-accepted):1–14. 10.1080/07391102.2020.1772885 PMC7284156 32448085

[18] Liu Z, Huang C, Fan K, Wei P, Chen H, Liu S, et al Virtual screening of novel noncovalent inhibitors for SARS-CoV 3 C-like proteinase. Journal of chemical information and modeling. 2005;45(1):10–7. 10.1021/ci049809b 15667124

[19] Mahanta S, Chowdhury P, Gogoi N, Goswami N, Borah D, Kumar R, et al Potential anti-viral activity of approved repurposed drug against main protease of SARS-CoV-2: an in silico based approach. Journal of Biomolecular Structure and Dynamics. 2020;(just-accepted):1–15. 10.1080/07391102.2020.1768902 32406317

[20] Khan SA, Zia K, Ashraf S, Uddin R, Ul-Haq Z. Identification of chymotrypsin-like protease inhibitors of SARS-CoV-2 via integrated computational approach. Journal of Biomolecular Structure and Dynamics. 2020:1–10. 10.1080/07391102.2020.1751298 32238094

[21] N Powers C, N Setzer W. An in-silico investigation of phytochemicals as antiviral agents against dengue fever. Combinatorial chemistry & high throughput screening. 2016;19(7):516–36. 27151482 10.2174/1386207319666160506123715 PMC5411999

[22] Falcone Ferreyra ML, Rius S, Casati P. Flavonoids: biosynthesis, biological functions, and biotechnological applications. Frontiers in plant science. 2012;3:222 10.3389/fpls.2012.00222 23060891 PMC3460232

[23] Leong CNA, Tako M, Hanashiro I, Tamaki H. Antioxidant flavonoid glycosides from the leaves of Ficus pumila L. Food Chemistry. 2008;109(2):415–20. 10.1016/j.foodchem.2007.12.069 26003366

[24] Kumar M, Ahmad A, Rawat P, Khan MF, Rasheed N, Gupta P, et al Antioxidant flavonoid glycosides from Evolvulus alsinoides. Fitoterapia. 2010;81(4):234–42. 10.1016/j.fitote.2009.09.003 19748554

[25] Wen L, Zhao Y, Jiang Y, Yu L, Zeng X, Yang J, et al Identification of a flavonoid C-glycoside as potent antioxidant. Free Radical Biology and Medicine. 2017;110:92–101. 10.1016/j.freeradbiomed.2017.05.027 28587909

[26] Bhardwaj VK, Singh R, Sharma J, Rajendran V, Purohit R, Kumar S. Identification of bioactive molecules from Tea plant as SARS-CoV-2 main protease inhibitors. Journal of Biomolecular Structure and Dynamics. 2020;(just-accepted):1–13. 10.1080/07391102.2020.1766572 PMC7256349 32397940

[27] Li S, Dong P, Wang J, Zhang J, Gu J, Wu X, et al Icariin, a natural flavonol glycoside, induces apoptosis in human hepatoma SMMC-7721 cells via a ROS/JNK-dependent mitochondrial pathway. Cancer letters. 2010;298(2):222–30. 10.1016/j.canlet.2010.07.009 20674153

[28] Li H, Huang J, Yang B, Xiang T, Yin X, Peng W, et al Mangiferin exerts antitumor activity in breast cancer cells by regulating matrix metalloproteinases, epithelial to mesenchymal transition, and β-catenin signaling pathway. Toxicology and applied pharmacology. 2013;272(1):180–90. 10.1016/j.taap.2013.05.011 23707762

[29] Sati P, Dhyani P, Bhatt ID, Pandey A. Ginkgo biloba flavonoid glycosides in antimicrobial perspective with reference to extraction method. Journal of traditional and complementary medicine. 2019;9(1):15–23. 10.1016/j.jtcme.2017.10.003 30671362 PMC6335473

[30] Tagousop CN, Ekom SE, Ngnokam D, Voutquenne-Nazabadioko L. Antimicrobial activities of flavonoid glycosides from Graptophyllum grandulosum and their mechanism of antibacterial action. BMC complementary and alternative medicine. 2018;18(1):1–10. 10.1186/s12906-017-2057-9 30219066 PMC6139119

[31] Tasdemir D, Kaiser M, Brun R, Yardley V, Schmidt TJ, Tosun F, et al Antitrypanosomal and antileishmanial activities of flavonoids and their analogues: in vitro, in vivo, structure-activity relationship, and quantitative structure-activity relationship studies. Antimicrobial agents and chemotherapy. 2006;50(4):1352–64. 10.1128/AAC.50.4.1352-1364.2006 16569852 PMC1426963

[32] Reutrakul V, Ningnuek N, Pohmakotr M, Yoosook C, Napaswad C, Kasisit J, et al Anti HIV-1 flavonoid glycosides from Ochna integerrima. Planta medica. 2007;73(07):683–8. 17562490 10.1055/s-2007-981538

[33] Xiao J, Capanoglu E, Jassbi AR, Miron A. Advance on the flavonoid C-glycosides and health benefits. Critical Reviews in Food Science and Nutrition. 2016;56(sup1):S29–S45. 26462718 10.1080/10408398.2015.1067595

[34] Khalil H, Abd El Maksoud AI, Roshdey T, El‐Masry S. Guava flavonoid glycosides prevent influenza A virus infection via rescue of P53 activity. Journal of medical virology. 2019;91(1):45–55. 10.1002/jmv.25295 30153335

[35] Xu G, Dou J, Zhang L, Guo Q, Zhou C. Inhibitory effects of baicalein on the influenza virus in vivo is determined by baicalin in the serum. Biological and Pharmaceutical Bulletin. 2010;33(2):238–43. 10.1248/bpb.33.238 20118546

[36] Dou J, Chen L, Xu G, Zhang L, Zhou H, Wang H, et al Effects of baicalein on Sendai virus in vivo are linked to serum baicalin and its inhibition of hemagglutinin-neuraminidase. Archives of virology. 2011;156(5):793–801. 10.1007/s00705-011-0917-z 21286764

[37] Wu W, Li R, Li X, He J, Jiang S, Liu S, et al Quercetin as an antiviral agent inhibits influenza A virus (IAV) entry. Viruses. 2016;8(1):6. 10.3390/v8010006 PMC4728566 26712783

[38] Tao J, Hu Q, Yang J, Li R, Li X, Lu C, et al In vitro anti-HIV and-HSV activity and safety of sodium rutin sulfate as a microbicide candidate. Antiviral research. 2007;75(3):227–33. 10.1016/j.antiviral.2007.03.008 17459492

[39] Nguyen TTH, Woo H-J, Kang H-K, Kim Y-M, Kim D-W, Ahn S-A, et al Flavonoid-mediated inhibition of SARS coronavirus 3C-like protease expressed in Pichia pastoris. Biotechnology letters. 2012;34(5):831–8. 10.1007/s10529-011-0845-8 22350287 PMC7087583

[40] Liu W, Morse JS, Lalonde T, Xu S. Learning from the past: possible urgent prevention and treatment options for severe acute respiratory infections caused by 2019‐nCoV. Chembiochem. 2020. 10.1002/cbic.202000047 PMC7162020 32022370

[41] Li F. Structure, function, and evolution of coronavirus spike proteins. Annual review of virology. 2016;3:237–61. 10.1146/annurev-virology-110615-042301 27578435 PMC5457962

[42] Wahedi HM, Ahmad S, Abbasi SW. Stilbene-based natural compounds as promising drug candidates against COVID-19. Journal of Biomolecular Structure and Dynamics. 2020;(just-accepted):1–16. 10.1080/07391102.2020.1762743 32345140

[43] Zhu L, George S, Schmidt MF, Al-Gharabli SI, Rademann J, Hilgenfeld R. Peptide aldehyde inhibitors challenge the substrate specificity of the SARS-coronavirus main protease. Antiviral research. 2011;92(2):204–12. 10.1016/j.antiviral.2011.08.001 21854807 PMC7114241

[44] Elmezayen AD, Al-Obaidi A, Şahin AT, Yelekçi K. Drug repurposing for coronavirus (COVID-19): in silico screening of known drugs against coronavirus 3CL hydrolase and protease enzymes. Journal of Biomolecular Structure and Dynamics. 2020:1–13. 10.1080/07391102.2020.1758791 PMC7189413 32306862

[45] Mittal L, Kumari A, Srivastava M, Singh M, Asthana S. Identification of potential molecules against COVID-19 main protease through structure-guided virtual screening approach. Journal of Biomolecular Structure and Dynamics. 2020;(just-accepted):1–26. 10.1080/07391102.2020.1768151 PMC7256355 32396769

[46] Pettersen EF, Goddard TD, Huang CC, Couch GS, Greenblatt DM, Meng EC, et al UCSF Chimera—a visualization system for exploratory research and analysis. Journal of computational chemistry. 2004;25(13):1605–12. 10.1002/jcc.20084 15264254

[47] Jin Z, Du X, Xu Y, Deng Y, Liu M, Zhao Y, et al Structure of M pro from SARS-CoV-2 and discovery of its inhibitors. Nature. 2020:1–5. 10.1038/s41586-020-2223-y 32272481

[48] Trott O, Olson AJ. AutoDock Vina: improving the speed and accuracy of docking with a new scoring function, efficient optimization, and multithreading. Journal of computational chemistry. 2010;31(2):455–61. 10.1002/jcc.21334 19499576 PMC3041641

[49] Kutzner C, Páll S, Fechner M, Esztermann A, de Groot BL, Grubmüller H. More bang for your buck: Improved use of GPU nodes for GROMACS 2018. Journal of computational chemistry. 2019;40(27):2418–31. 10.1002/jcc.26011 31260119

[50] Abraham MJ, Murtola T, Schulz R, Páll S, Smith JC, Hess B, et al GROMACS: High performance molecular simulations through multi-level parallelism from laptops to supercomputers. SoftwareX. 2015;1:19–25.

[51] Benson NC, Daggett V. A comparison of multiscale methods for the analysis of molecular dynamics simulations. The Journal of Physical Chemistry B. 2012;116(29):8722–31. 10.1021/jp302103t 22494262 PMC3406285

[52] Xiao J. Dietary flavonoid aglycones and their glycosides: Which show better biological significance? Critical reviews in food science and nutrition. 2017;57(9):1874–905. 10.1080/10408398.2015.1032400 26176651

[53] Valentová K, Vrba J, Bancířová M, Ulrichová J, Křen V. Isoquercitrin: pharmacology, toxicology, and metabolism. Food and Chemical Toxicology. 2014;68:267–82. 10.1016/j.fct.2014.03.018 24680690

[54] Amaretti A, Raimondi S, Leonardi A, Quartieri A, Rossi M. Hydrolysis of the rutinose-conjugates flavonoids rutin and hesperidin by the gut microbiota and bifidobacteria. Nutrients. 2015;7(4):2788–800. 10.3390/nu7042788 25875120 PMC4425173

[55] Serseg T, Benarous K, Yousfi M. Hispidin and Lepidine E: two Natural Compounds and Folic acid as Potential Inhibitors of 2019-novel coronavirus Main Protease (2019-nCoVMpro), molecular docking and SAR study. Current computer-aided drug design. 2020. 10.2174/1573409916666200422075440 32321407

[56] Khan A, Ali SS, Khan MT, Saleem S, Ali A, Suleman M, et al Combined drug repurposing and virtual screening strategies with molecular dynamics simulation identified potent inhibitors for SARS-CoV-2 main protease (3CLpro). Journal of Biomolecular Structure and Dynamics. 2020:1–12. 10.1080/07391102.2020.1779128 PMC7309305 32552361

[57] Anand K, Ziebuhr J, Wadhwani P, Mesters JR, Hilgenfeld R. Coronavirus main proteinase (3CLpro) structure: basis for design of anti-SARS drugs. Science. 2003;300(5626):1763–7. 10.1126/science.1085658 12746549

[58] Gupta S, Singh AK, Kushwaha PP, Prajapati KS, Shuaib M, Senapati S, et al Identification of potential natural inhibitors of SARS-CoV2 main protease by molecular docking and simulation studies. Journal of Biomolecular Structure and Dynamics. 2020;(just-accepted):1–19. 10.1080/07391102.2020.1776157 PMC7312383 32476576

[59] Kumar V, Dhanjal JK, Kaul SC, Wadhwa R, Sundar D. Withanone and caffeic acid phenethyl ester are predicted to interact with main protease (Mpro) of SARS-CoV-2 and inhibit its activity. Journal of Biomolecular Structure and Dynamics. 2020;(just-accepted):1–17. 10.1080/07391102.2020.1772108 PMC7284143 32431217

[60] Khan A, Khan M, Saleem S, Babar Z, Ali A, Khan AA, et al Phylogenetic analysis and structural perspectives of RNA-dependent RNA-polymerase inhibition from SARs-CoV-2 with natural products. Interdisciplinary Sciences: Computational Life Sciences. 2020;12(3):335–48. 10.1007/s12539-020-00381-9 PMC7332347 32617855

