# Supplementary material for: Potential bioactive glycosylated flavonoids as SARS-CoV-2 main protease inhibitors: A molecular docking and simulation studies
Source: PLoS One. 2020 Oct 15;15(10):e0240653. doi: 10.1371/journal.pone.0240653 (PMC7561147; doi:10.1371/journal.pone.0240653)
Supplement: S1 Fig — Kinetic and potential energies for Mpro complex with (a), Quercetin -3 Rhamnose (b) Myricetin 3-rutinoside and (c) Rutin for 50 ns. (DOCX) [file pone.0240653.s001.docx]

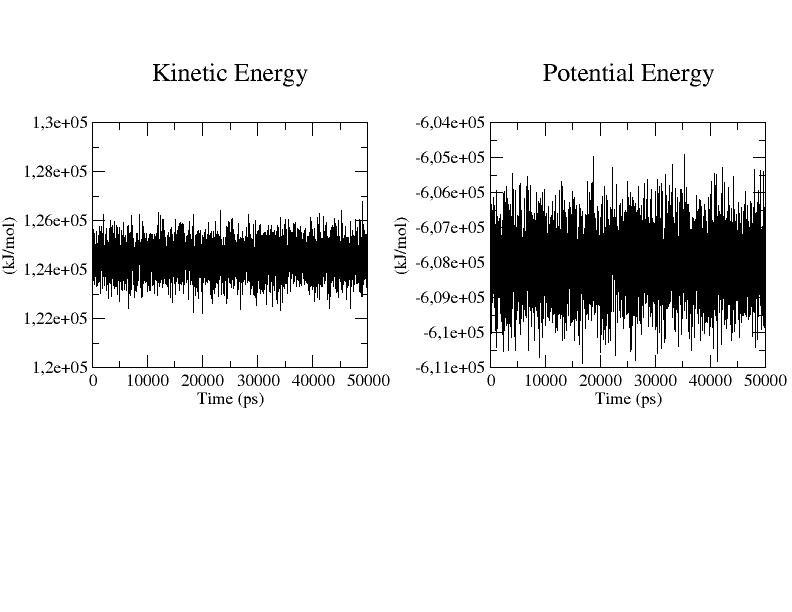


b)

a)

c)


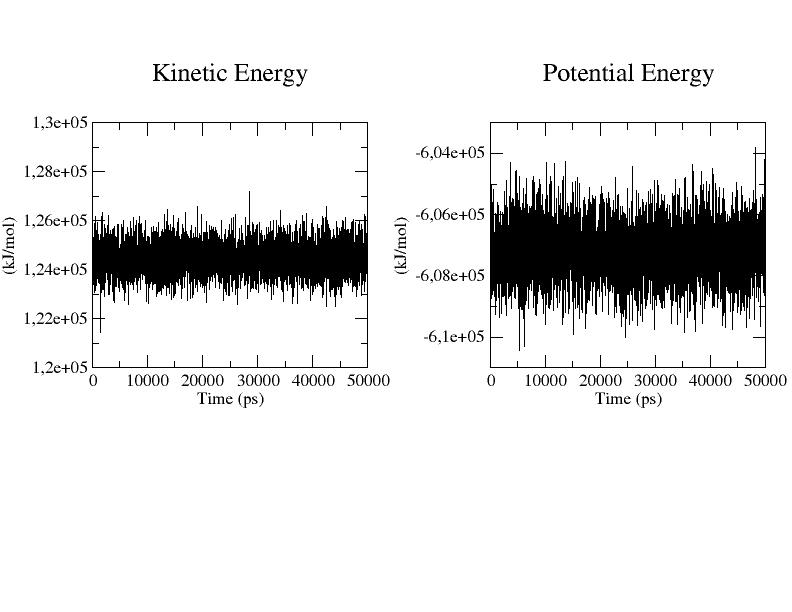


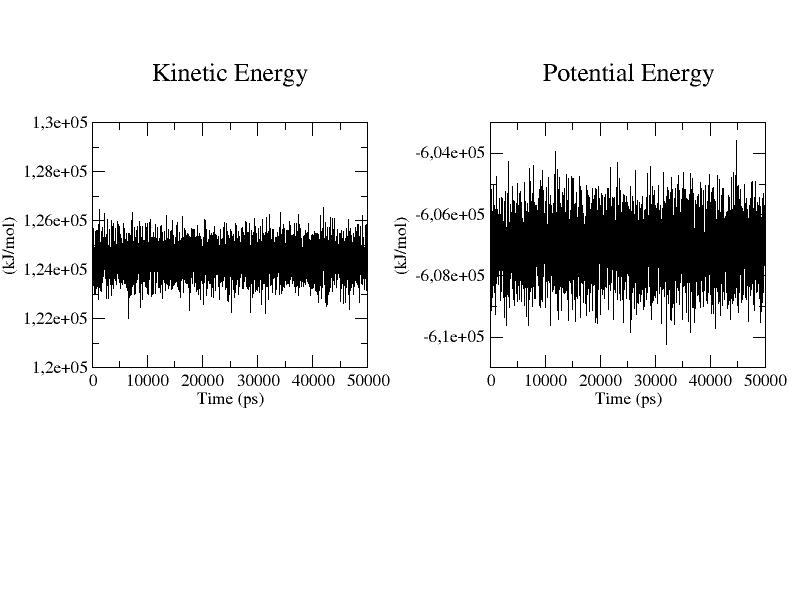


Supplementary material : Kinetic and potential energies for Mpro complex with(a), Quercetin -3 Rhamnose (b)Myricetin 3-rutinoside and (c) Rutin for 50 ns
